# Multiple local recurrences of primary sternal chondrosarcoma: tumor manipulation or self-seeding

**DOI:** 10.1186/s13019-023-02213-5

**Published:** 2023-04-08

**Authors:** Riad Abdel Jalil, Farah A. Abdallah

**Affiliations:** 1 Department of Research, King Hussein Cancer Center, Queen Rania Al Abdullah Street, Amman, 11941 Jordan; 2 Department of Thoracic Oncology, King Hussein Cancer Center, Amman, Jordan

**Keywords:** Chondrosarcoma, Sternum, Local recurrence, Seeding

## Abstract

**Background:**

Primary sternal chondrosarcoma, although rare, is the most common malignant tumor of the sternum. The gold standard treatment is complete surgical excision, which frequently causes the instability of the thorax necessitating future reconstruction. Local recurrence is common increasing the risk of distant metastasis.

**Case presentation:**

A 60-year-old male patient was diagnosed with primary sternum chondrosarcoma and underwent surgical excision with negative resection margins. Later, he was found to have two local recurrences at 11 months and 37 months post initial excision. The two recurrences were surgically removed followed by local adjuvant radiation.

**Conclusion:**

The seeding theories have been reported more frequently with relation to diagnostic biopsy procedures, tumor manipulation and self-seeding tumors. The patient developed two local recurrences despite total resection with negative margins, without concerns regarding seeding in distant metastasis.

## Introduction

The thorax consists of bone and soft tissue, protects the internal organs and supports the process of respiration. The tumors found most often in the chest wall are usually metastases from other organs such as breast, kidney or lung. In contrast, primary tumors of the chest wall constitute an average of 0.2–2% [1].The histopathology of the primary tumors found in the chest wall is represented by chondrosarcoma in 20% of the cases, of which 20% originate in the sternum [1].

Sternal chondrosarcoma is usually resistant to chemotherapy and radiotheraphy, the only valid alternative being the surgical treatment [2]. The surgical treatment may cause chest wall instability and requires reconstruction [3].

In case of high-grade tumors, inadequately resected margins or surgical intervention performed in hospitals that are not experienced with tumors, local metastases occur in up to 50% of patients who undergo curative surgery [1, 4].

Local recurrence is an indicator of systemic metastasis and a poor outcome. The specialty literature has reported that 29% of patients who have local recurrence will develop distant metastasis [5]. Thus, all patients with resected tumors must be followed both clinically and imaging by chest computed tomography (CT) every three to six months in the first five years then annually for at least ten [6].

A 60-year-old male was diagnosed with primary sternal chondrosarcoma and underwent surgical treatment, removing completely the tumor, but two local metastases were discovered four years after the primary intervention without systemic metastases.In the current specialized literature no such case has been presented.

## Case presentation

**Fig. 1:**
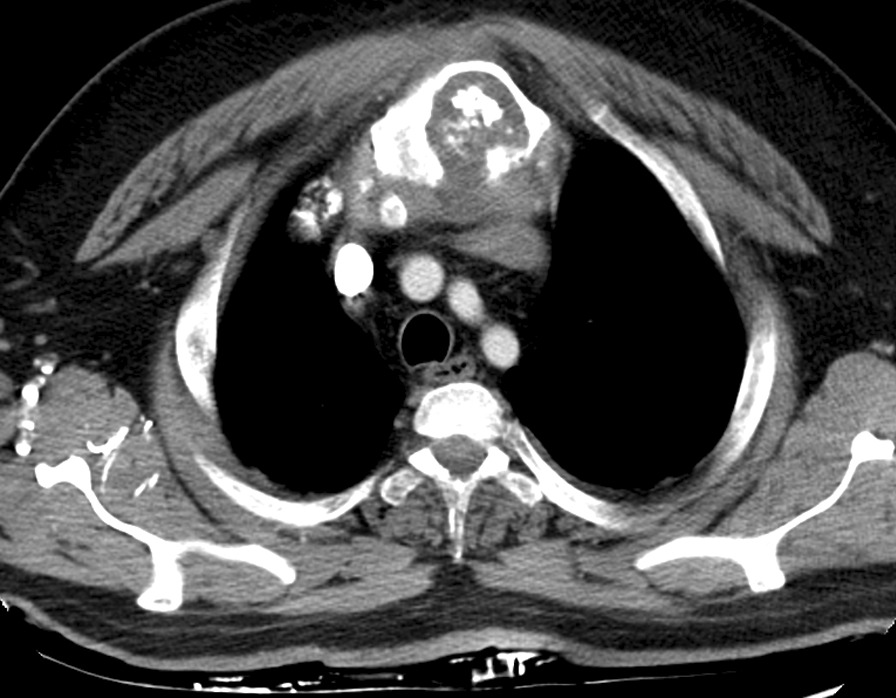
Pre-operative computed tomography (CT) of anterior chest wall chondrosarcoma arising from the sternum with internal calcification

A 60-year-old male patient, known to have hypertension, diabetes mellitus, and a history of cardiac catheterization and cholecystectomy, was referred to the hospital with painful enlarging swelling of the anterior chest. The patient also developed at that time progressive shortness of breath and a productive cough. He denied any history of relevant trauma to the area. The physical examination revealed a hard anterior chest mass measuring 8 × 8 × 8 cm. The abdomen was soft and lax with no palpable organomegaly. Chest computed tomography (CT) showed an extensive, destructive lesion involving the whole manubrium with a soft tissue component of 8 × 8 × 6 cm. The destructive osseous lesion with internal calcification in the sternum was associated with an anterior parasternal soft tissue component and another posterior intrathoracic state intermediate soft tissue component, which was abutting with the left brachiocephalic vein (Fig. 1). There was evidence of a few bilateral non-specific pulmonary lesions. The patient underwent three cycles of chemotherapy without response. Findings raised the possibility of a primary bone tumor without definite pulmonary metastasis. Chondrosarcoma was a top differential. Positron emission tomography PET-CT demonstrated a heterogeneous mildly hypermetabolic (low FDG-avid) large expansive destructive manubrial mass with intrathoracic (superior anterior mediastinal) and extrathoracic soft tissue components. This was compatible with the primary chondrosarcoma. Biopsy showed grade II chondrosarcoma. Therefore, the patient was referred to a neoplasm specialized center, King Hussein Cancer Center.

**Fig. 2:**
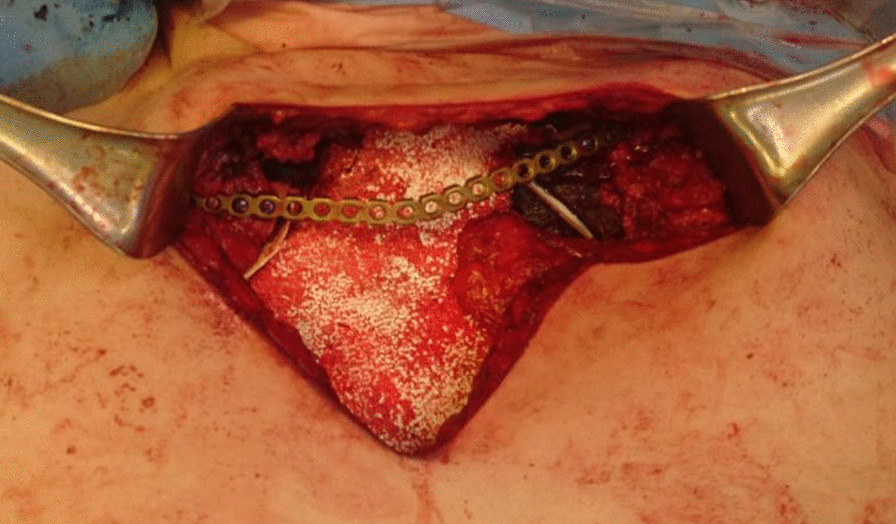
The chest wall constructed with the non-absorbable mesh, one titanium plate connecting the right and left clavicles to avoid clavicle retraction and to maintain chest wall convexity

**Fig. 3:**
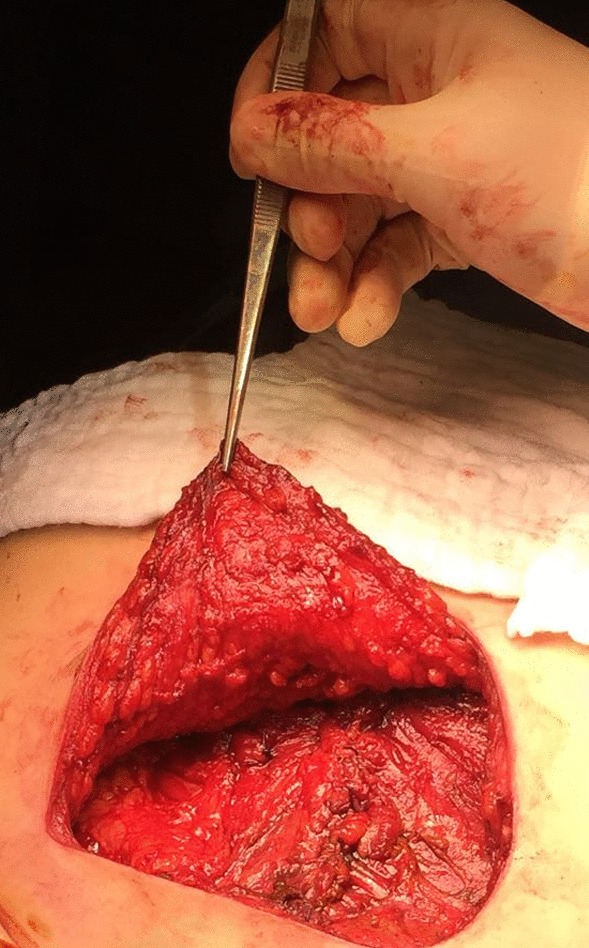
Bilateral pectoralis major advancement muscle flaps were used to cover the reconstruction area

Following the conclusion of the multidisciplinary team, the surgical treatment was decided. Resection included the upper half of the sternum en bloc with medial parts of both clavicles and the central portion of the first and second ribs bilaterally. The defect was reconstructed with a non-absorbable mesh which is the most important and strongest part of the reconstruction; one titanium plate was added to connect the right and left clavicles together in order to avoid their retraction and to maintain the anterior superior chest wall convexity shape (Fig. 2). The plate was fixed with screws as well as non-absorbable stitches through the plate holes to the clavicles to prevent rotation or dislodgement. The mesh and the plate were covered with bilateral pectoralis major advancement muscle flaps (Fig. 3). The patient was fully informed about the necessity of strict movement instruction to keep his clavicles in the adduction position and to avoid forceful movements like pushups which may cause plate fracture. The patient was discharged on the ninth day post-operative with favorable evolution.

Intra-operative histopathology showed a completely resected 7.5 × 5 × 4 cm tumor with 2–3 cm free margin—a grade II chondrosarcoma with 30% necrosis.

At three weeks post-operatively, the patient presented to the emergency department with pain where the surgical intervention was. It was found a fracture of the titanium plate between the clavicles due to excessive hard work and was admitted for plate revision and fixation with stainless steel wires.

The patient remained asymptomatic with non-remarkable CT images during all follow-up visits, until eleven months later when he presented with swelling of the left chest. CT showed a mass in the left chest wall in the subcutaneous area, no recurrence in the sternum, and no lung lesions. Biopsy confirmed recurrent grade II chondrosarcoma with positive S-100, negative Pan-Ck immunostain, and inconclusive IDH-1. The patient then underwent left chest wall mass wide local resection. The post-operative course was favorable and the patient was discharged on the third day. Histopathology exam confirmed a recurrent well defined grade II chondrosarcoma measuring 11 × 10.5 × 7 cm with up to 2 cm free, but close margin due to the recurrence location near the lateral aspect of the clavicle.So post-operative radiation followed.

After twenty-six months after resection of the first recurrence, a CT scan showed a new lobulated mildly enhancing mass in the right upper anterior chest wal, just above the medial end of the right clavicle suspicious of recurrent disease (confirmed by biopsy). A whole-body CT scan showed no distant metastases. Surgery was the next step with right upper chest wall mass resection en bloc with skin and major pectoralis muscle beside foreign body titanium plate removal. The post-operative course was uneventful. Pathological examination confirmed grade II chondrosarcoma, measuring 4.5 × 3 × 2 cm, mitotic rate 1/10, and necrosis with up to 2 cm completefree margin at the lateral aspect. Random biopsies lateral to the resected tumor were obtained, despite no masses encountered there and then sent to histopathology, which proved positive for chondrosarcoma. The patient was discussed in the sarcoma multidisciplinary team for adjuvant radiotherapy.

## Discussion

Chondrosarcoma is the most common primary malignant bone tumor of the chest wall [7]. It has a higher incidence in males between the third and sixth decade of life. Presenting signs and symptoms vary, most commonly painful swelling, but can also be painless swelling, cough, paresthesia, chest trauma history in the location of the tumor, irradiation history to the chest wall, or incidental finding on thoracic imaging [8].

Imaging evaluation for any suspected chest wall mass is the chest CT with contrast in setting up a therapeutic plan since the bone and soft tissue window outline tumor characteristics and invasion, while pulmonary window help evaluates lung metastasis [6]. The CT images indicate a well-defined, lobulated soft tissue mass with foci of chondroid matrix ossification (resulting from the destruction of the cortex and mineralization of tumor matrix) [9, 10]. Magnetic resonance image (MRI) is not mandatory for diagnosis [6]. Meanwhile, PET helps differentiate benign cartilaginous tumors from chondrosarcoma, aside the ability to rule out extrapulmonary metastases [6]. Histopathology is the certainty diagnosis, excisional biopsy for small tumors and incisional biopsy reserved for large resectable tumors (more than 4 cm) [10, 11].

Non-excisional biopsy increases the risk of tumor implantation along the needle track after aspiration biopsy and in the soft tissue after open biopsy thus it is discouraged [10, 11].

Surgery is the treatment of choice, aiming for wide aggressive free margin radical en bloc excision with chest wall reconstruction [6]. Complete resection and grade of tumors are the most important prognostic factors predicting survival [5].

Chest wall chondrosarcoma shows aggressiveness due to it is high risk of local recurrence and metastasis despite negative mergines resection [4]. Rascoe et al., reported a local recurrence in 50% of widely excised chondrosarcoma [6]. In addition to Lenze et al. who found that 50% of locally recurrent chondrosarcoma had a clear resection margins [12]. Moreover, local recurrence increased the risk of distant metastasis (*p* = 0.004) [5].

In some cases, it is hard to establish whether the local recurrence is due to spontaneous tumor spreading, needle tract seeding or due to tumor manipulation [13].

Needle tract seeding is defined as tumor cell implantation by a needle while biopsy or examine the tumor, the risk of this iatrogenic phenomenon has increasingly been reported in the last few decades, some believe that needle tract implantation may change the tumor grade and the operability of the tumor [14]. The prevalence is hard to estimate as many of the cases may be underreported and many patients may lose follow-up. The time between the aspiration to implantation detection can be as early as months up to four years, poorly differentiated and high-grade tumors tend to have earlier implantation [15]. Close follow-up will help detect any implantations earlier.Despite all, Saghieh et all,reported the very low probability of needle tract seeding in bone sarcoma [16]. On the other hand, tumor self-seeding is another theory that was first brought out to publication in 2009 [17]. Which exhibit that tumor cells that leave the primary tumor or so-called circulating tumor cells CTCs can spread to not only distant sites but also back to the primary tumor sites, where they can find no preferable environment to habituate. Tumor inflammatory cytokines from the primary site like IL-6 and IL-8 attract CTCs and leaky tumor neovasculature allows it to leak back into their primary site, where it secrets MMP-collagenase-1 and CXCL1 which stimulates tumor growth and angiogenesis [18]. Gross et all,reported a self- seeding osteosarcoma by chemotaxis and IL-6 production [19]. Beside that, the seeding of local tissue during resection is theoretically possible.

After all, Local recurrence decrease survival rates to 45% at 5 and 10 years compared to 89% and 81% at 5 and 10 years in patients without recurrence [12].

In our case, the patient had two separate local recurrences despite previous resection with free margins in the primary tumor surgical resection and the first recurrence resection. Last surgery revealed the presence of tumor cells in random biopsies lateral to the tumor whose margin was free of tumor upon microscopic examination despite no apparent masses which may indicate seeding of the tumor either with self-seeding or during manipulation of the tumor in the previous surgeries.

## Conclusion

Chest wall chondrosarcoma can relapses locally. Local recurrence is usually accompanied by distant metastasis. Controlling primary tumors with free resection margins is the main factor to prevent a recurrence but not definite. In this case, two instance of local recurrences have been demonstrated in four years despite free margins resection and the lack of systemic metastasis, which raise a concern of tumor seeding, and most likely due to tumor manipulation or self seeding tumors. Seeding is a raw theory and we encourage more studies and experments in this field to help understand the definite mechanism and approaching treatment manners.

## Data Availability

Not applicable.

## Abbreviations

CT: Computed tomography
PET: Positron emission tomography
MRI: Magnetic resonance image
CTCs: Circulating tumor cells
